# A basic community dynamics experiment: Disentangling deterministic and stochastic processes in structuring ecological communities

**DOI:** 10.1002/ece3.9568

**Published:** 2022-12-04

**Authors:** Mark Davidson Jewell, Graham Bell

**Affiliations:** ^1^ Department of Biology McGill University Montreal Quebec Canada; ^2^ Redpath Museum McGill University Montreal Quebec Canada

**Keywords:** community dynamics, ecological drift, Lemnaceae, neutral, niche, species coexistence

## Abstract

Community dynamics are governed by two opposed processes: species sorting, which produces deterministic dynamics leading to an equilibrium state, and ecological drift, which produces stochastic dynamics. Despite a great deal of theoretical and empirical work aiming to demonstrate the predominance of one or the other of these processes, the importance of drift in structuring communities and maintaining species diversity remains contested. Here, we present the results of a basic community dynamics experiment using floating aquatic plants, designed to measure the relative contributions of species sorting and ecological drift to community change over about a dozen generations. We found that species sorting became overwhelmingly dominant as the experiment progressed, and directed communities toward a stable equilibrium state maintained by negative frequency‐dependent selection. The dynamics of any particular species depended on how far its initial frequency was from its equilibrium frequency, however, and consequently the balance of sorting and drift varied among species.

## Graphical abstract

Community dynamics are governed by two opposed processes: species sorting, which produces deterministic dynamics leading to an equilibrium state, and ecological drift, which produces stochastic dynamics. Here, we report a basic ecological dynamics experiment using floating aquatic plants designed to quantify the relative contributions of these processes to community change.

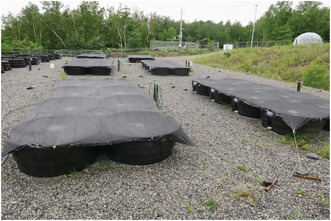

## 1 INTRODUCTION

Patterns in the composition and diversity of species in a community are the result of many interacting processes. Borrowing concepts from population genetics, Vellend (2010, 2016) distilled these down to four fundamental processes in a conceptual synthesis of community ecology: selection, ecological drift, speciation, and dispersal. Since speciation and dispersal are responsible for the introduction of new variation into the community, the dynamics of a closed community is essentially governed by selection (also referred to as species sorting) and ecological drift alone. Species sorting is the equivalent of natural selection at the level of species, which will produce distinct assemblages of species in different habitats, each local community consisting of those species best adapted to their local conditions of growth. This classical “niche‐based” view asserts that species coexistence is due to functional differences between species and predicts deterministic dynamics. Ecological drift is the equivalent of genetic drift at the level of species, which will produce a distinctive assemblage of species in any given place whose composition is unrelated to local conditions. This “neutral‐based” view assumes the functional equivalency of species and predicts stochastic community dynamics. The relative importance of these two processes in structuring communities has been vigorously debated in the last two decades and many attempts have been made to show that one of these processes is much more important than the other (Hubbell, 2006; Rosindell et al., 2011; Wennekes et al., 2012; Wright, 2002). It would be difficult to support either extreme view, however, because both processes will be active at all times everywhere, and the main goal of community ecology should be to understand how the balance between them depends on the underlying physical and biotic characteristics of sites. Many recent developments have been proposed to resolve the niche‐neutral controversy (Adler et al., 2007; Chase, 2014; Fisher & Mehta, 2014; Haegeman & Loreau, 2011; Leibold & McPeek, 2006; Matthews & Whittaker, 2014; Shoemaker et al., 2020; Siqueira et al., 2020), and there is a growing body of empirical experimental work aimed at disentangling stochastic from deterministic processes in community assembly (Chase, 2010; Gilbert & Levine, 2017; Ron et al., 2018).

Species sorting and ecological drift will have directly opposed effects on the species composition of communities under a given set of environmental conditions. Under species sorting, communities that initially differ in species abundances will converge toward the same composition, which represents the stable equilibrium community for that set of conditions (Jablonski, 2008). Under ecological drift, communities that are initially identical in species abundances will diverge in composition over time (Gilbert & Levine, 2017). The relative contributions of these two processes to community dynamics (changes over time in species composition) can therefore be estimated by setting up replicated communities with different initial composition. Ecological drift will cause divergence of replicate communities of any given initial composition. Species sorting will cause convergence of communities that initially differ in composition.

A third factor which might influence how a community changes over time is its initial state, as both drift and sorting may be historically contingent (Chase, 2003; Fukami, 2015). A species' initial frequency may influence its sensitivity to ecological drift due to the tendency of stochasticity to increase in importance in smaller effective populations. Likewise, species sorting might depend on a mechanism that favored abundant species, such as priority effects or growth inhibition by exudates. The contributions of sorting, drift, and initial state will sum to the overall change in composition observed over a given period of time.

Such experiments have been done for single‐species populations to estimate the contributions of natural selection, genetic drift, and ancestry to the evolution of fitness and of phenotypes such as cell size in bacteria and the evolution of heterotrophy in *Chlamydomonas* (Bell, 2013; Travisano et al., 1995). Despite the clear analogy of these processes in population genetics to community ecology (Vellend, 2010), no similar work has yet been done in multi‐species communities. Here, we extend experimental evolution into ecology to estimate the relative contributions of species sorting (the ecological equivalent of natural selection), ecological drift (genetic drift), and initial state (ancestry) to community species dynamics.

We assembled experimental communities of floating aquatic macrophytes from the family *Lemnaceae* that frequently coexist in the field. These are highly reduced angiosperms that consist of a single leaf‐like frond which may or may not bear a submerged unbranched root, depending on the species. Reproduction is nearly always asexual and vegetative, which results in extremely short generation times of less than a week in eutrophic conditions. Many species are widespread and abundant in lentic ecosystems and often coexist in multi‐species communities consisting of hundreds of thousands to millions of individuals. Because of their small size and short generation time, they are being increasingly used as a model system in ecology and evolution (Hart et al., 2019; Laird & Barks, 2018; Vu et al., 2019) and enable us to run highly replicated experiments lasting more than a dozen generations in a single season. Here, we report the results of a basic community dynamics experiment using replicated semi‐natural communities consisting of four such species of *Lemnaceae*. By manipulating initial relative abundances of species and following changes in species composition over time, we can quantify community divergence and convergence. The main goal of this experiment was to estimate the relative contributions of species sorting, ecological drift, and initial state to community change.

## 2 MATERIALS AND METHODS

### 2.1 Source community

The source plant community was isolated from a eutrophic pond adjacent to fallowed agricultural fields on the Macdonald campus of McGill University, Quebec, Canada (45°42′N, 73°94′W). The pond sustains a diverse community of floating macrophytes, the four most abundant being *Lemna minor* (Lm), *Lemna trisulca* (Lt), *Spirodela polyrhiza* (Sp), *and Wolffia columbiana* (Wc), all in the family *Lemnaceae*. Large samples consisting of hundreds of thousands of individuals were taken in June 2020 and manually separated into the constituent species.

### 2.2 Experimental design

**FIGURE 1:**
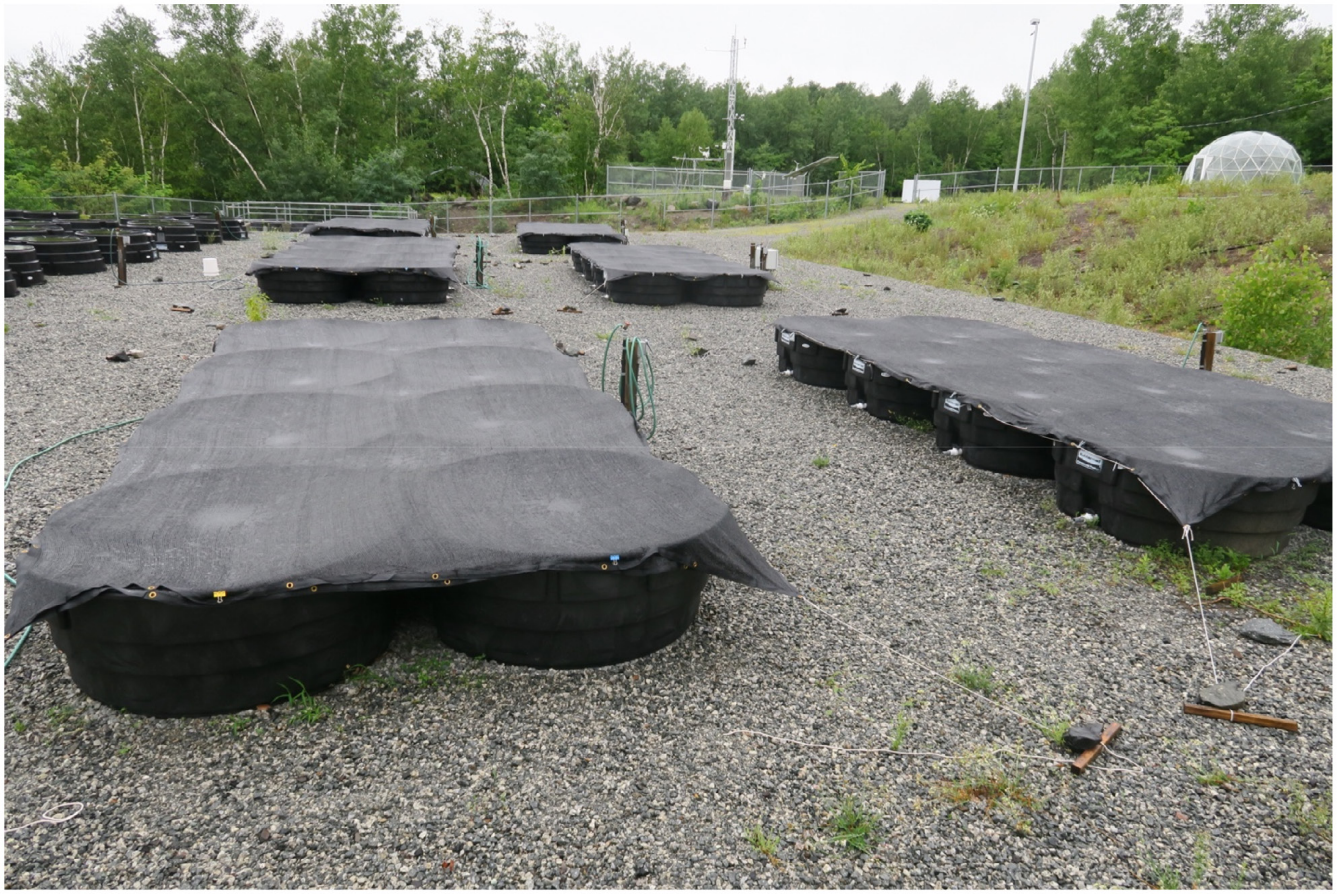
The experimental setup. Forty‐eight mesocosms arranged in six blocks of eight on the LEAP platform at McGill's Gault Nature Reserve. Each block is covered with shade cloth which serves to mimic canopy cover and minimize the input of debris by wind.

The experiment was conducted at the LEAP (Large Experimental Array of Ponds) facility at Gault Nature Reserve of McGill University in Quebec, Canada (45°32′N, 73°08′W; Fugère et al., 2020; Figure 1). Forty‐eight large mesocosms (surface area = 2.43 m^2^) were filled each with 500 L of water piped from Lac Hertel, a mesotrophic lake on the reserve, 1 km upstream of the experiment. The water was sieved to remove fish, tadpoles, macroinvertebrates, and macrophytes, but contained intact communities of zooplankton and phytoplankton. The removal of these larger organisms was to decrease unwanted variation due to sampling. Material from the source community was used to assemble four community types defined by the initial relative abundance of each species (10%, 20%, 30%, or 40%; Table 1). Relative abundance was calculated as mass‐weighted frequencies using an average value of individual mass for each species. Each community was seeded with a total of 1 g wet mass of community biomass, which works out to between ~2000–3000 individuals, depending on the community type (Table 1). Abundances of the larger species were determined by manual counting, while Wc, only ~0.5 mm wide, was weighed and added in bulk, using an estimate of mean frond mass. Initial community densities translated to roughly 5%–10% surface cover. Communities were mixed after inoculation to remove any initial spatial variation. Each of the four community types were replicated in 12 mesocosms (total number of mesocosms = 48) which were arranged in six blocks of eight mesocosms, each block containing two replicates of each community type, with community type randomized within block. All mesocosms received a one‐time initial addition of inorganic Nitrogen and Phosphorus (KNO_3_ and H_2_KPO_4_), to obtain initial dissolved concentrations of these nutrients in the mesocosms comparable to those of the pond from which the source community was taken (800 μg L^−1^ N and 40 μg L^−1^ P). The mesocosms were covered with 70% shade cloth to mimic canopy cover. Although this minimized the input of wind‐carried debris like leaf litter, rainwater could pass through the mesh cloth, which roughly balanced water lost due to evaporation. Communities were then left to grow for 12 weeks, from the beginning of July to the end of September, ending shortly before the first frost. All mesocosms were randomly sampled every 2 weeks to estimate species relative abundances. This was done by first mixing the communities to break up species clustering (Hart et al., 2019; Jewell & Bell, 2022), then removing a fixed percentage of the surface area (~5%) with a net. Although mixing eliminated communities' spatial structure, it allowed us to efficiently obtain representative samples. These samples were exhaustively counted before being returned to the mesocosm.

**TABLE 1:** Initial composition of the four community types by mass‐weighted relative abundances of the four species: *Lemna minor* (Lm), *Lemna trisulca* (Lt), *Spirodela polyrhiza* (Sp), and *Wolffia columbiana* (Wc).

Community type	Species	Initial relative abundances (%)	Initial no. of individuals
A	*Lm*	40	1081
	*Lt*	30	150
	*Sp*	20	72
	*Wc*	10	625
	Total	100	1928
B	*Lm*	30	811
	*Lt*	40	200
	*Sp*	10	36
	*Wc*	20	1250
	Total	100	2297
C	*Lm*	20	541
	*Lt*	10	50
	*Sp*	40	145
	*Wc*	30	1875
	Total	100	2611
D	*Lm*	10	270
	*Lt*	20	100
	*Sp*	30	109
	*Wc*	40	2500
	Total	100	2979

### 2.3 Statistical analysis

The main goal of this experiment was to estimate the contributions of species sorting, ecological drift, and initial state to community change. Overall variation in final species composition among communities can be broken into these three components, whose contributions to variation can be partitioned using an ANOVA framework (Bell, 2013; Travisano et al., 1995). If *Y*_*ij*_ is the final frequency of the focal species in community type *i* and replicate *j*, then its deviation from that species' mean initial frequency, *Y*_initial_, can be partitioned into three additive components representing the three sources of variation:

$$Y_{\mathrm{ij}}-Y_{\text{initial}}=\left(Y_{\mathrm{ij}}-Y_{i}\right)+\left(Y_{i}-Y\right)+\left(Y-Y_{\text{initial}}\right)$$

where *Y*_*i*_ is the mean final frequency of the focal species in community type *i*, and *Y* is the grand mean final frequency of the focal species across all community types and replicates. For *n* community types (communities with different initial species composition) each replicated m times, the total variation attributable to sorting, drift, and initial state can be calculated as follows:

- nm S (*Y* − *Y*_initial_)^2^, the shift in grand mean representing an overall convergence to an equilibrium composition (sorting),
- m S (*Y*_*i*_ − *Y*)^2^, the variance among community types around the grand mean representing the influence of a community's initial state, and
- S S (*Y*_*ij*_ − *Y*_*i*_)^2^, the variance among replicates of same community type representing neutral variation (drift).

Such a partition was done for each species at the end of the experiment to estimate the overall contributions of sorting, drift, and initial state, as well as for each intermediate census to assess how the contributions changed over time.

We calculated a normalized value of the change in relative abundance of each species at each census in each mesocosm as the difference between the relative abundance of that species in the current and immediately preceding census, divided by its relative abundance in the preceding census. For each species, we used the regression of this normalized change in relative abundance on its preceding relative abundance to determine whether species dynamics were frequency‐dependent. We used the *X*‐intercept (at which change in relative abundance is zero) as an estimate of the equilibrium abundance of that species in a stable community.

## 3 RESULTS

**FIGURE 2:**
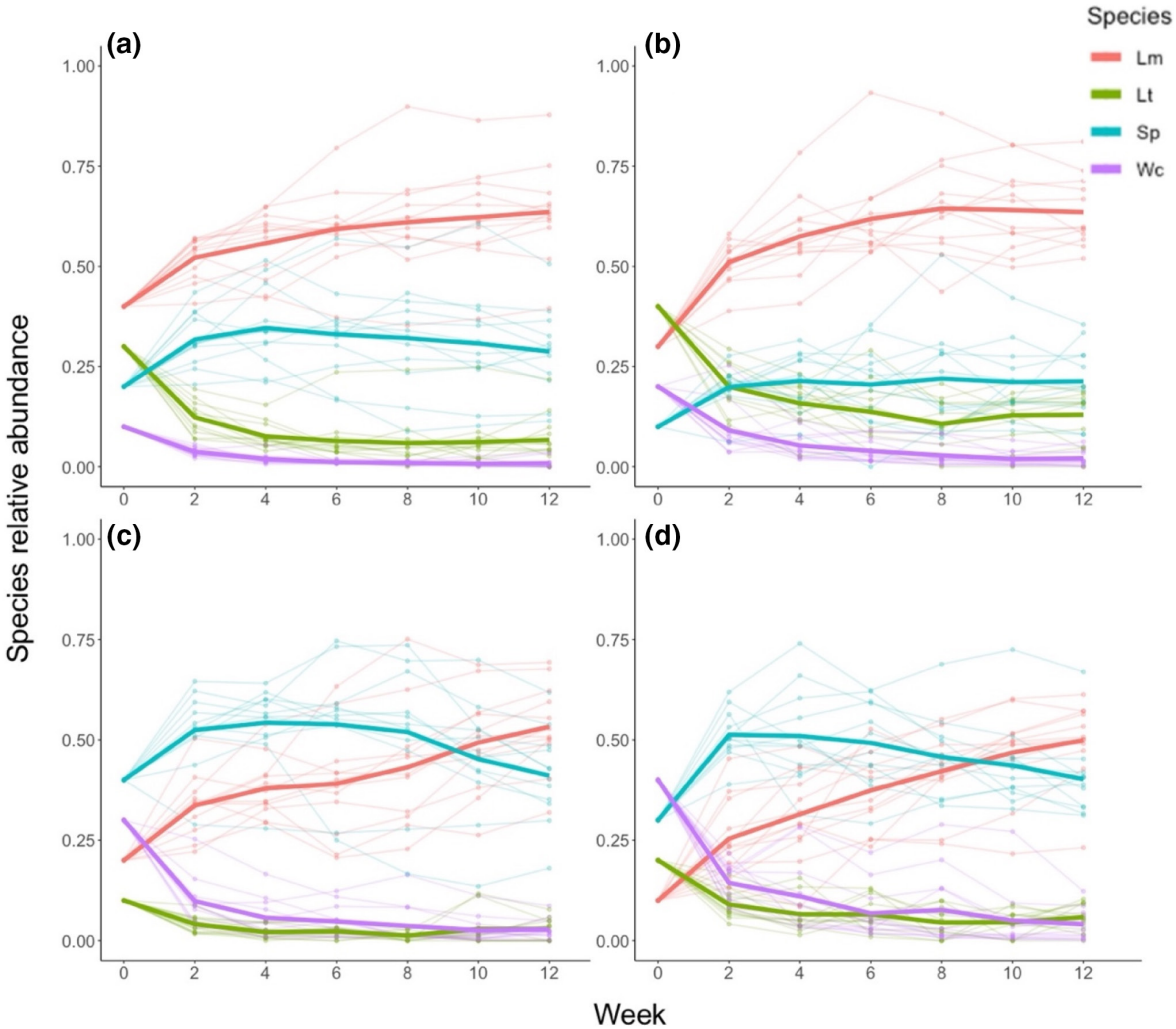
Community dynamics over 12 weeks of growth. All four community types (a–d) consist of the same four species, which differ in their initial relative abundances depending on the community type (Table 1). The four species were *L. minor* (Lm), *L. trisulca* (Lt), *S. polyrhiza* (Sp), and *W. columbiana* (Wc). Each community type was replicated in 12 mesocosms, the means of which are shown as bold lines.

There was an initial sharp drop in the relative abundances of *Lemna trisulca* (Lt) and *Wolffia columbiana* (Wc) because of transfer shock, with a fraction of plants immediately sinking to the bottom of the mesocosms. Because of this, we have used week 2 as the initial time point. Similar transfer shocks have been observed in other experiments with many of the same species (Jewell & Bell, 2022). After this initial settling time, community composition continued to change during the 12‐week experiment with *Lemna minor* (Lm) coming to dominate most communities, regardless of its initial frequency, at the expense of Lt and Wc which became rare (but not extinct) in most communities (Figure 2). *Spirodela polyrhiza* (Sp), maintained moderate abundances in most communities. The overall trajectory of community composition seemed to be largely independent of initial composition and was consistent among replicates. This was the case regardless of whether relative abundance was weighted by mass (as for the main analysis), or left as un‐weighted individual counts (Figures S1 and S2).

**FIGURE 3:**
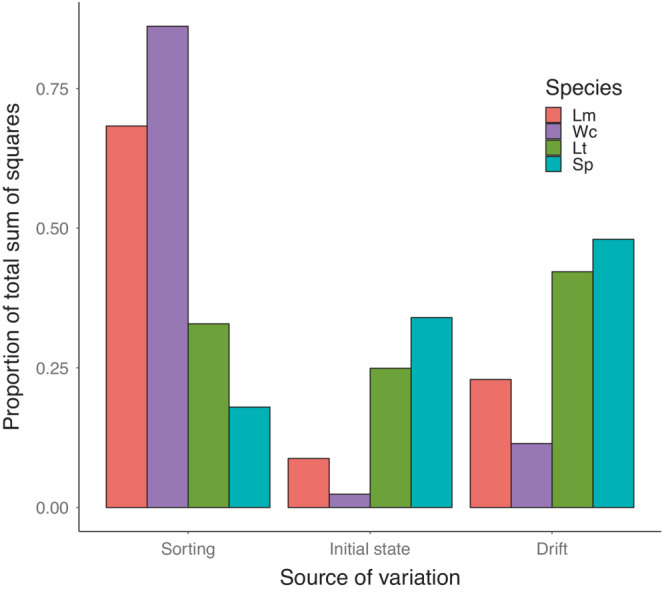
Contributions of sorting, drift, and initial state as proportions of the total sum of squares to overall community change. Contributions were calculated separately for each of the four species: *L. minor* (Lm), *W. columbiana* (Wc), *L. trisulca* (Lt), and *S. polyrhiza* (Sp). The underlying ANOVA table is in Appendix S1.

**FIGURE 4:**
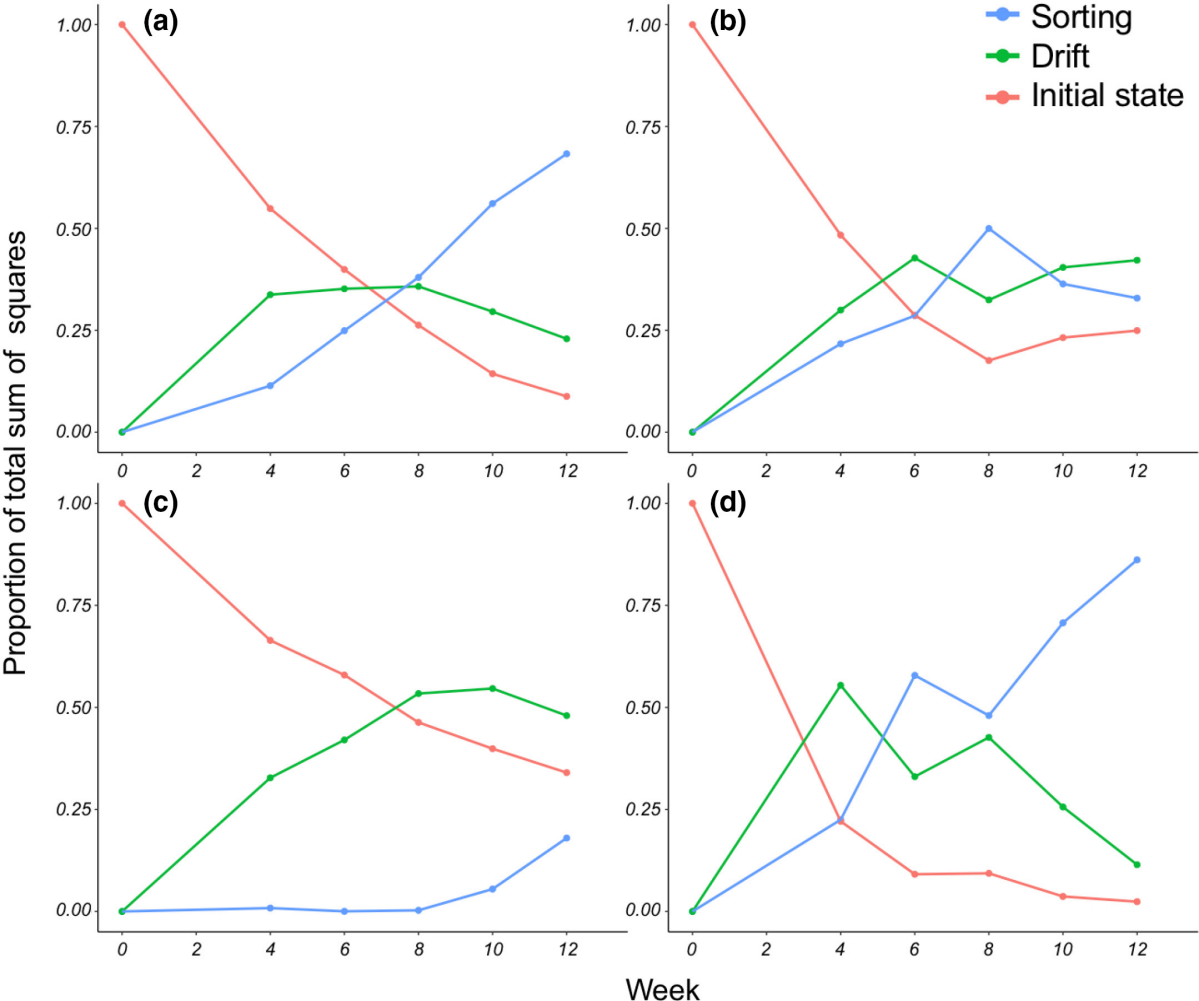
Contributions, measured as proportion of total sum of squares, of sorting, initial state, and drift to overall community change over the course of the experiment for (a) *L. minor*, (b) *L. trisulca*, (c) *S. polyrhiza*, and (d) W. columbiana. Sums of squares are from the ANOVA shown in Appendix S1.

By the end of the experiment, each community type had diverged substantially from its initial composition. The extent of this divergence has three components: sorting as the source of directional change, drift as idiosyncratic divergence among replicates, and initial state as retention of differences among community types. To quantify the relative contributions of sorting, drift, and initial state to final community composition we partitioned the overall sum of squares into components representing these three processes (Figure 3). This analysis was done for each time point (see Appendix S1), to evaluate how these three sources of community change varied over the course of the experiment (Figure 4).

**FIGURE 5:**
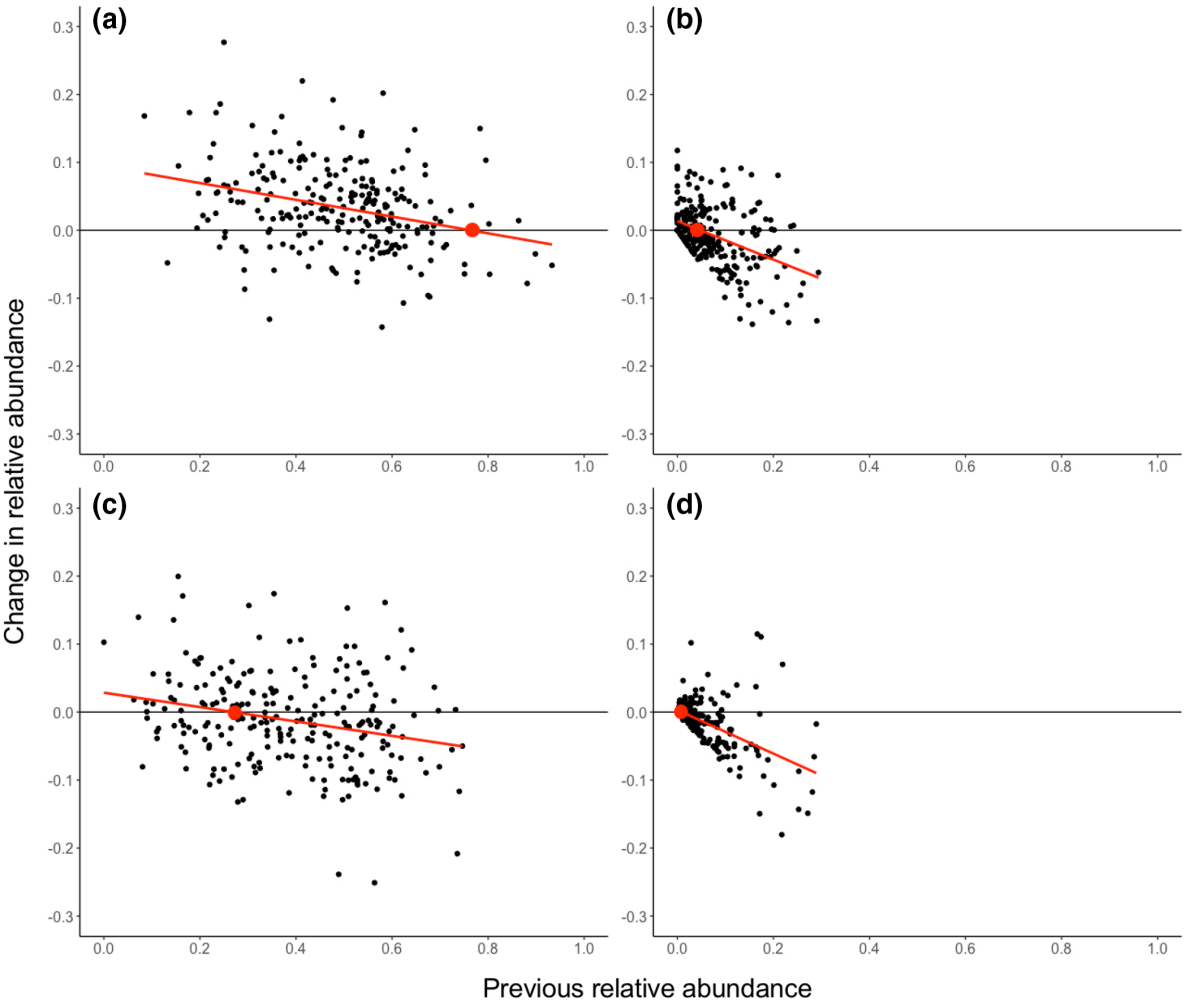
Change in species relative abundance as a function of previous relative abundance for (a) *L. minor*, (b) *L. trisulca*, (c) *S. polyrhiza*, and (d) *W. columbiana*. *Y* = 0 indicates no change in species relative abundance and thus where the regression line intersects this gives an estimate for the equilibrium frequency of that species.

We regressed the normalized change in relative abundance between time points against previous relative abundance for each species to obtain estimates of equilibrium species frequencies (Figure 5). These regressions are autocorrelated, but they can be used to estimate the equilibrium frequency of a species as the *X*‐intercept, yielding 0.76 for Lm, 0.05 for Lt, 0.27 for Sp, and 0.01 for Wc. When adjusted to sum to 1, these frequencies are 0.70 for Lm, 0.04 for Lt, 0.25 for Sp, and 0.01 for Wc.

## 4 DISCUSSION

By the end of the experiment, the initial state of the community had ceased to be a principal determinant of composition for all species (Figure 3). A large contribution of initial state would indicate either that species frequencies had not changed, or that change was contingent on history, either through drift (e.g., because of the increased risk of extinction when a species becomes rare) or sorting (e.g., through facilitation, inhibition, or competitive intransitivity). The dominant process depended on the focal species, as the dynamics of Lm and Wc were governed predominantly by sorting, while those of Lt and Sp were governed largely by drift (Figure 3). Overall, Lm benefited at the expense of Wc, while Lt and Sp remained largely static with a moderate amount of stochastic variation around their mean state. A modest contribution of sorting (and therefore a large relative contribution of drift) could be caused either by weak competition or because the equilibrium frequency of a species fell close to its average initial frequency over all community types (as was the case for Sp, whose initial and equilibrium relative abundances were both 0.25). Conversely, the dynamics of the species whose equilibrium frequencies are the furthest from their average initial frequencies (Lm and Wc) were most dominated by species sorting.

The strong effect of sorting and the absence of any strong effect of initial state suggests that the community tends toward an equilibrium composition, either through competitive exclusion or stable coexistence. Given a relatively constant and spatially homogeneous environment (Chesson, 2017; May, 1973), the distance from equilibrium which an actual community lies is determined by the balance of sorting and drift. Whether or not this equilibrium community involves the stable coexistence of several species can be determined by considering how the frequency of a species changes as a function of its current value (Figure 5). The presence of a large negative correlation for all four species is evidence of negative frequency‐dependent selection acting as a stable coexistence mechanism (Adler et al., 2007; Chesson, 2000). The slope of this correlation indicates the strength of the frequency dependence, and its intercept with zero change in frequency indicates the equilibrium frequency of that species. This negative frequency dependence is probably a widespread coexistence mechanism in natural communities of floating freshwater macrophytes (Armitage & Jones, 2019; Barrat‐Segretain & Elger, 2004; Gérard & Triest, 2018; Hart et al., 2019) responsible for maintaining local diversity. Any real community will deviate from the ideal equilibrium composition through ecological drift.

The balance of sorting, drift, and initial state shifted in a simple and predictable way over time. At the beginning of the experiment, since no change has yet occurred, all variation in community composition is due to initial state (Figure 4). As time progresses, the relative contribution of initial state diminishes for all species, indicating the lessening contribution of initial state to community dynamics. Both species sorting and ecological drift increase in importance, and as the community nears equilibrium, roughly balance each other. This balance is however species‐specific, due to the strength of competition. The further away a species' frequency is from its equilibrium value, the stronger species sorting will act to bring it closer. Thus, both sorting and drift are acting on all species at all times, but how they combine depends on the species and will be determined by its competitive advantage and how far it is from its equilibrium frequency.

**FIGURE 6:**
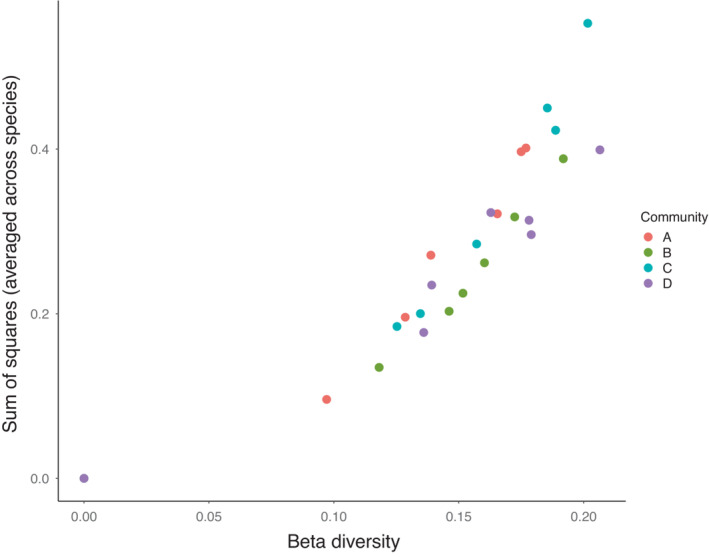
A comparison of two measures of ecological drift. Sums of squares were calculated for each community type, time point, and species, and then averaged across the four species to allow comparison with beta diversity. Beta diversity was calculated as mean pairwise Bray–Curtis pairwise dissimilarity for each community type at each time point.

The standard approach to quantify stochastic effects on community structure is with the use of pairwise beta‐diversity indices (e.g., Bray–Curtis; Anderson et al., 2011). Compositional dissimilarity is calculated for all pairwise combinations of sites in the same conditions and averaged to produce an estimate of among site (beta) diversity due to stochasticity, ranging from 0 to 1 (Gilbert & Levine, 2017; Ron et al., 2018). Our approach of using an ANOVA framework allows us to partition the contribution stochastic processes into those due to ecological drift (variation in vital rates among individuals of the same species) and priority effects (stochastic variation in order of colonization), and allows us to compare these numerically with the contribution from species sorting. Furthermore, obtaining estimates for each species separately can reveal how these fundamental processes may operate distinctly for different species in a community. The weakness of such an approach is that the contribution of sorting is highly sensitive to the initial frequencies (and their distances from the equilibrium frequencies). Consequently, estimates of the proportion of the total sum of squares due to stochasticity will also be sensitive to an arbitrary initial frequency. To work around this, it may also be informative to consider the contributions of stochastic processes as the raw within‐group (drift) and among‐group (initial state) sums of squares. We compared these measures to the more commonly used Bray–Curtis beta‐diversity, calculated among replicate communities of each community type, at each time point, and found that both methods captured largely the same variability in species frequencies (Figure 6). This among‐replicate variability, quantified either as a sum of squares or Bray–Curtis beta‐diversity, will include any unintentional biotic and abiotic variation among replicate mesocosms that feed back to influence species vital rates. That any effect of imperfect replication is included in our estimates of ecological drift further highlights the relative importance of species sorting in our system.

We conclude that the dynamics of our experimental communities shifted over time but were eventually dominated by species sorting, which resulted in the predictable and largely deterministic shift toward an equilibrium state. The contribution of initial state declined consistently over time, but the balance between species sorting and ecological drift varied among species because, as in any community, some species were closer than others to their equilibrium frequency.

## AUTHOR CONTRIBUTIONS

**Mark Davidson Jewell:** Conceptualization (supporting); data curation (lead); formal analysis (equal); funding acquisition (equal); investigation (equal); methodology (equal); writing – original draft (lead); writing – review and editing (lead). **Graham Bell:** Conceptualization (lead); formal analysis (equal); funding acquisition (equal); investigation (equal); methodology (equal); project administration (lead); writing – review and editing (supporting).

## CONFLICT OF INTEREST

The authors declare no competing interests.

## Supporting information


Appendix S1

## Data Availability

Data is available on dryad doi: https://doi.org/10.5061/dryad.15dv41p1m.
