# Supplementary material for: A basic community dynamics experiment: Disentangling deterministic and stochastic processes in structuring ecological communities
Source: Ecol Evol. 2022 Dec 4;12(12):e9568. doi: 10.1002/ece3.9568 (PMC9720002; doi:10.1002/ece3.9568)
Supplement: Supplementary file 1 — Appendix S1 [file ECE3-12-e9568-s001.docx]

**Supporting information**


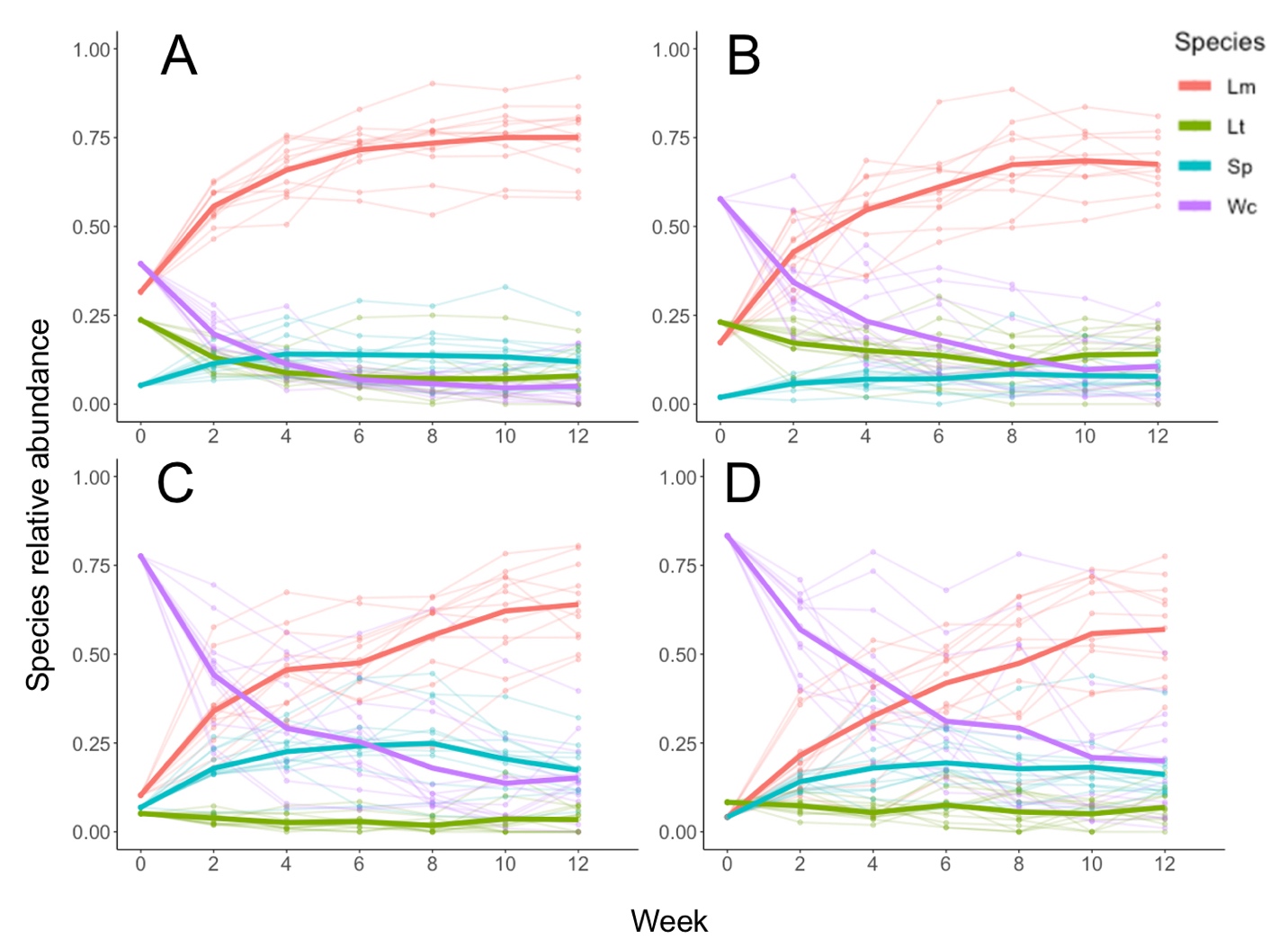


Fig. S1. *Community dynamics over 12 weeks of growth. Here, species relative abundance is measured as proportion of total individuals in the community, and is not weighted by species average mass. All four community types (A, B, C & D) consist of the same four species, which differ in their initial relative abundances depending on the community type (Table 1). The four species were L. minor (Lm), L. trisulca (Lt), S. polyrhiza (Sp), and W. columbiana (Wc). Each community type was replicated in 12 mesocosms, the means of which are shown as bold lines.*


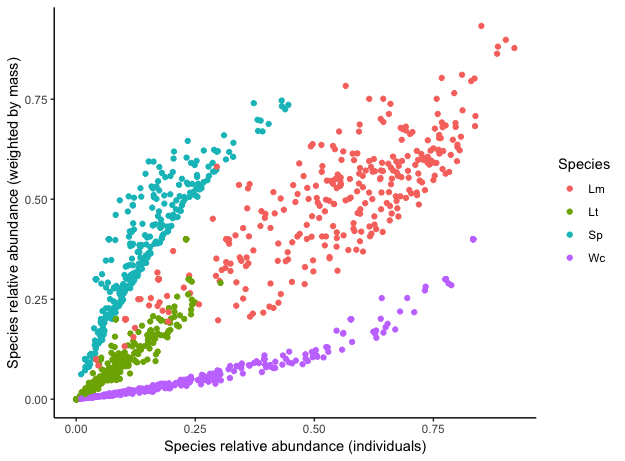


*Fig. S2. A comparison of two measures of species relative abundance, weighted and unweighted by species average mass. Points included all data from the experiment (all community types, replicates and species).*

*Appendix S1. ANOVA tables. The partition of variance in change in species frequency into components representing sorting, drift, and initial state. Analyses are done for each species separately, and for each time point over the course of the experiment.*

| Week: 4  Species: Lm | | | | |
| --- | --- | --- | --- | --- |
| Source | ***Degrees of freedom*** | ***Sum of squares*** | ***Mean Square*** | ***Variance component*** |
| Sorting | *1* | *0.125* | *0.125* | *0* |
| Initial state | *3* | *0.603* | *0.201* | *0.016* |
| Drift | *44* | *0.371* | *0.008* | *0.008* |
| Total | *48* | *1.099* | *0.023* |  |

| Week: 4  Species: Lt | | | | |
| --- | --- | --- | --- | --- |
| Source | ***Degrees of freedom*** | ***Sum of squares*** | ***Mean Square*** | ***Variance component*** |
| Sorting | *1* | *0.053* | *0.053* | *0.000* |
| Initial state | *3* | *0.118* | *0.039* | *0.003* |
| Drift | *44* | *0.073* | *0.002* | *0.002* |
| Total | *48* | *0.243* | *0.005* |  |

| Week: 4  Species: Sp | | | | |
| --- | --- | --- | --- | --- |
| Source | ***Degrees of freedom*** | ***Sum of squares*** | ***Mean Square*** | ***Variance component*** |
| Sorting | *1* | *0.010* | *0.010* | *0* |
| Initial state | *3* | *0.837* | *0.279* | *0.022* |
| Drift | *44* | *0.412* | *0.009* | *0.009* |
| Total | *48* | *1.260* | *0.026* |  |

| Week: 4  Species: Wc | | | | |
| --- | --- | --- | --- | --- |
| Source | ***Degrees of freedom*** | ***Sum of squares*** | ***Mean Square*** | ***Variance component*** |
| Sorting | *1* | *0.051* | *0.051* | *0.001* |
| Initial state | *3* | *0.050* | *0.017* | *0.001* |
| Drift | *44* | *0.126* | *0.003* | *0.003* |
| Total | *48* | *0.228* | *0.005* |  |

| Week: 6  Species: Lm | | | | |
| --- | --- | --- | --- | --- |
| Source | ***Degrees of freedom*** | ***Sum of squares*** | ***Mean Square*** | ***Variance component*** |
| Sorting | *1* | *0.377* | *0.377* | *0.004* |
| Initial state | *3* | *0.604* | *0.201* | *0.016* |
| Drift | *44* | *0.532* | *0.012* | *0.012* |
| Total | *48* | *1.512* | *0.032* |  |

| Week: 6  Species: Lt | | | | |
| --- | --- | --- | --- | --- |
| Source | ***Degrees of freedom*** | ***Sum of squares*** | ***Mean Square*** | ***Variance component*** |
| Sorting | *1* | *0.080* | *0.080* | *0.001* |
| Initial state | *3* | *0.081* | *0.027* | *0.002* |
| Drift | *44* | *0.120* | *0.003* | *0.003* |
| Total | *48* | *0.281* | *0.006* |  |

| Week: 6  Species: Sp | | | | |
| --- | --- | --- | --- | --- |
| Source | ***Degrees of freedom*** | ***Sum of squares*** | ***Mean Square*** | ***Variance component*** |
| Sorting | *1* | *0.001* | *0.001* | *0* |
| Initial state | *3* | *0.843* | *0.281* | *0.022* |
| Drift | *44* | *0.612* | *0.014* | *0.014* |
| Total | *48* | *1.455* | *0.030* |  |

| Week: 6  Species: Wc | | | | |
| --- | --- | --- | --- | --- |
| Source | ***Degrees of freedom*** | ***Sum of squares*** | ***Mean Square*** | ***Variance component*** |
| Sorting | *1* | *0.125* | *0.125* | *0.002* |
| Initial state | *3* | *0.020* | *0.007* | *0.000* |
| Drift | *44* | *0.071* | *0.002* | *0.002* |
| Total | *48* | *0.215* | *0.004* |  |

| Week: 8  Species: Lm | | | | |
| --- | --- | --- | --- | --- |
| Source | ***Degrees of freedom*** | ***Sum of squares*** | ***Mean Square*** | ***Variance component*** |
| Sorting | *1* | *0.708* | *0.708* | *0.011* |
| Initial state | *3* | *0.489* | *0.163* | *0.012* |
| Drift | *44* | *0.667* | *0.015* | *0.015* |
| Total | *48* | *1.864* | *0.039* |  |

| Week: 8  Species: Lt | | | | |
| --- | --- | --- | --- | --- |
| Source | ***Degrees of freedom*** | ***Sum of squares*** | ***Mean Square*** | ***Variance component*** |
| Sorting | *1* | *0.159* | *0.159* | *0.003* |
| Initial state | *3* | *0.056* | *0.019* | *0.001* |
| Drift | *44* | *0.103* | *0.002* | *0.002* |
| Total | *48* | *0.318* | *0.007* | *0.00* |

| Week: 8  Species: Sp | | | | | |
| --- | --- | --- | --- | --- | --- |
| Source | ***Degrees of freedom*** | ***Sum of squares*** | ***Mean Square*** | ***Variance component*** | |
| Sorting | *1* | *0.004* | *0.004* | | *0* |
| Initial state | *3* | *0.654* | *0.218* | | *0.017* |
| Drift | *44* | *0.754* | *0.017* | | *0.017* |
| Total | *48* | *1.412* | *0.029* | |  |

| Week: 8  Species: Wc | | | | |
| --- | --- | --- | --- | --- |
| Source | ***Degrees of freedom*** | ***Sum of squares*** | ***Mean Square*** | ***Variance component*** |
| Sorting | *1* | *0.144* | *0.144* | *0.003* |
| Initial state | *3* | *0.028* | *0.009* | *0.001* |
| Drift | *44* | *0.128* | *0.003* | *0.003* |
| Total | *48* | *0.301* | *0.006* |  |

| Week: 10  Species: Lm | | | | |
| --- | --- | --- | --- | --- |
| Source | ***Degrees of freedom*** | ***Sum of squares*** | ***Mean Square*** | ***Variance component*** |
| Sorting | *1* | *1.089* | *1.089* | *0.021* |
| Initial state | *3* | *0.278* | *0.093* | *0.007* |
| Drift | *44* | *0.574* | *0.013* | *0.013* |
| Total | *48* | *1.942* | *0.040* |  |

| Week: 10  Species: Lt | | | | |
| --- | --- | --- | --- | --- |
| Source | ***Degrees of freedom*** | ***Sum of squares*** | ***Mean Square*** | ***Variance component*** |
| Sorting | *1* | *0.107* | *0.107* | *0.002* |
| Initial state | *3* | *0.068* | *0.023* | *0.002* |
| Drift | *44* | *0.119* | *0.003* | *0.003* |
| Total | *48* | *0.295* | *0.006* |  |

| Week: 10  Species: Sp | | | | |
| --- | --- | --- | --- | --- |
| Source | ***Degrees of freedom*** | ***Sum of squares*** | ***Mean Square*** | ***Variance component*** |
| Sorting | *1* | *0.064* | *0.064* | *0* |
| Initial state | *3* | *0.466* | *0.155* | *0.012* |
| Drift | *44* | *0.638* | *0.014* | *0.014* |
| Total | *48* | *1.168* | *0.024* |  |

| Week: 10  Species: Wc | | | | |
| --- | --- | --- | --- | --- |
| Source | ***Degrees of freedom*** | ***Sum of squares*** | ***Mean Square*** | ***Variance component*** |
| Sorting | *1* | *0.214* | *0.214* | *0.004* |
| Initial state | *3* | *0.011* | *0.004* | *0.000* |
| Drift | *44* | *0.078* | *0.002* | *0.002* |
| Total | *48* | *0.303* | *0.006* |  |

| Week: 12  Species: Lm | | | | |
| --- | --- | --- | --- | --- |
| Source | ***Degrees of freedom*** | ***Sum of squares*** | ***Mean Square*** | ***Variance component*** |
| Sorting | *1* | *1.389* | *1.389* | *0.028* |
| Initial state | *3* | *0.179* | *0.060* | *0.004* |
| Drift | *44* | *0.466* | *0.011* | *0.011* |
| Total | *48* | *2.034* | *0.042* |  |

| Week: 12  Species: Lt | | | | |
| --- | --- | --- | --- | --- |
| Source | ***Degrees of freedom*** | ***Sum of squares*** | ***Mean Square*** | ***Variance component*** |
| Sorting | *1* | *0.086* | *0.086* | *0.001* |
| Initial state | *3* | *0.066* | *0.022* | *0.002* |
| Drift | *44* | *0.111* | *0.003* | *0.003* |
| Total | *48* | *0.263* | *0.005* |  |

| Week: 12  Species: Sp | | | | | |
| --- | --- | --- | --- | --- | --- |
| Source | ***Degrees of freedom*** | ***Sum of squares*** | ***Mean Square*** | ***Variance component*** | |
| Sorting | *1* | *0.172* | *0.172* | | *0.001* |
| Initial state | *3* | *0.326* | *0.109* | | *0.008* |
| Drift | *44* | *0.460* | *0.010* | | *0.010* |
| Total | *48* | *0.958* | *0.020* | |  |

| Week: 12  Species: Wc | | | | |
| --- | --- | --- | --- | --- |
| Source | ***Degrees of freedom*** | ***Sum of squares*** | ***Mean Square*** | ***Variance component*** |
| Sorting | *1* | *0.220* | *0.220* | *0.005* |
| Initial state | *3* | *0.006* | *0.002* | *0.000* |
| Drift | *44* | *0.029* | *0.001* | *0.001* |
| Total | *48* | *0.256* | *0.005* |  |
